# Supplementary material for: Mass Balance and Compositional Analysis of Biomass Outputs from Cacao Fruits
Source: Molecules. 2022 Jun 9;27(12):3717. doi: 10.3390/molecules27123717 (PMC9254743; doi:10.3390/molecules27123717)
Supplement: Supplementary file 1 [file molecules-27-03717-s001.zip › molecules-1695546-supplementary.pdf]

SUPPLEMENTARY INFORMATION

Mass balance and compositional analysis of biomass outputs from cacao fruits

Marisol Vergara-Mendoza, Genny R. Martínez, Cristian Blanco-Tirado and Marianny Y. Combariza

Escuela de Química, Universidad Industrial de Santander, Bucaramanga 680002, Colombia

* marianny@uis.edu.co *To whom correspondence should be addressed.*

Supplementary information for Figure 2

Table S1. Traditional fresh cacao fruit biomass outputs: Cacao Husk (CH), Cacao Placenta (CP), and Cacao Beans (CBs) derived from the first stage of cacao fruit processing.

Load/Date	**Biomass outputs**			
	Fresh cacao husks (CH)	Fresh cacao placenta (CP)	Fresh cacao beans (CBs)	Losses
	wt %	wt %	wt %	wt %
February	70.13	2.48	22.68	4.71
March	67.84	2.61	23.64	5.92
April	67	2.80	25.24	4.97
May	66.1	2.82	27.33	3.75
June	63.71	2.33	24.28	9.69
July	70.55	2.48	26.72	0.25
August	67.09	2.17	24.21	6.54
September	69.09	2.49	22.12	6.32
October	67.87	2.83	23.89	5.42
November	66.33	2.64	25.69	5.36
December	60.1	2.48	24.17	13.25
January	67.66	2.85	24.57	4.93
Average + STDEV	66.96 ± 2.83	2.58 ± 0.22	24.55 ± 1.51	5.92 ± 3.15

Supplementary information for Figure 3

Table S2. Potential cacao fruit biomass outputs. dried cacao husk (CH). dried cacao placenta (CP). dried cacao beans (CBs). and cacao mucilage exudate (CME) derived from the second stage of cacao fruit processing.

Cacao load date	Biomass outputs			
	Cacao husks (CH)	Cacao placenta (CP)	Cacao beans (CB)	Cacao mucilage exudate (CME)
	Dried wt %	Dried wt %	Dried wt %	wt %
February	10.96	0.55	9.37	3.97
March	10.25	0.57	8.67	4.00
April	8.66	0.62	8.68	4.82
May	8.79	0.62	8.94	5.38
June	8.69	0.51	8.87	4.41
July	8.67	0.54	8.23	3.01
August	8.20	0.48	10.12	3.87
September	8.57	0.55	8.87	3.52
October	7.87	0.62	8.67	3.87
November	8.94	0.58	8.77	4.31
December	9.36	0.54	9.12	2.97
January	8.03	0.63	8.09	5.48
Average + STDEV	8.92 ± 0.90	0.57 ± 0.05	8.87 ± 0.52	4.13 ± 0.80

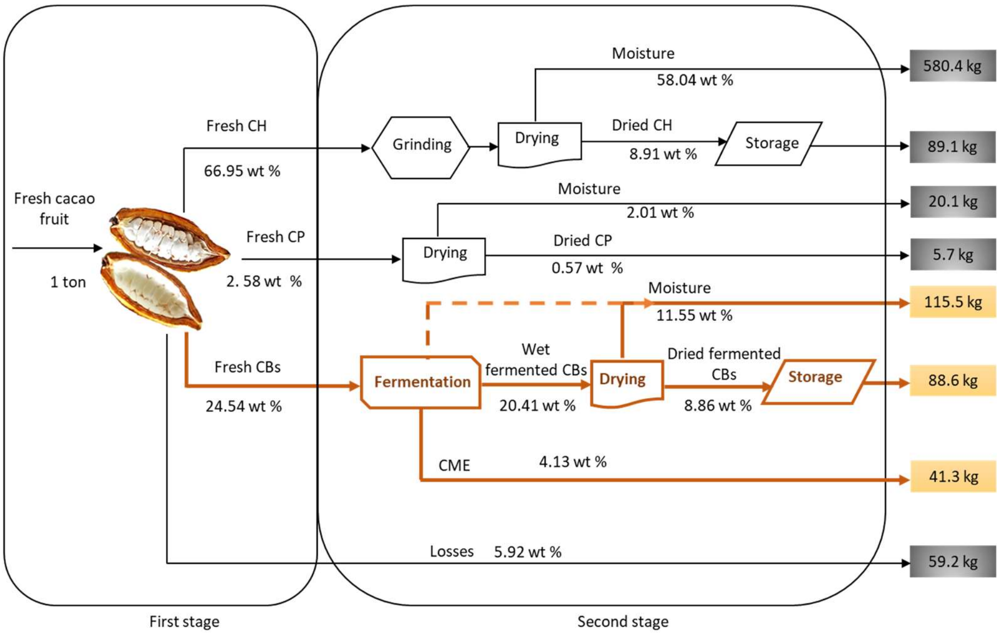

Figure S1. Supplementary Information with mass balance for the first and second stages of cacao fruit processing with averaged wt % data for all biomass outputs.

Supplementary information for Figure 5

Table S3. Composition analysis by load for fresh CH.

Cacao load date	Moisture	Total solids	Crude Fat	Crude Fiber	Crude protein	Ash	Total nitrogen	Lignin	Cellulose	Hemicellu	Calorific value
						wt %					(MJ kg $^{-1}$)
February	85.12	14.88	0.28	32.48	3.95	8.43	0.63	28.89	26.20	21.69	1.41
March	84.19	15.81	0.26	32.75	5.85	8.78	0.93	29.97	25.51	21.93	1.39
April	86.05	13.95	0.28	31.88	4.73	9.61	0.86	27.80	26.89	21.45	1.42
May	83.49	16.51	0.30	32.21	5.53	12.73	0.88	25.15	22.97	23.82	1.36
June	80.18	19.82	0.22	34.48	5.36	10.69	0.86	25.14	21.84	20.87	1.49
July	86.57	13.43	0.37	29.94	4.25	11.43	0.68	39.03	24.28	21.10	1.23
August	82.34	17.66	0.23	29.54	6.54	9.91	0.84	35.04	23.91	19.75	1.74
September	87.36	12.64	0.23	31.27	5.72	14.01	0.91	33.68	21.88	18.72	1.19
October	85.97	14.03	0.28	29.69	5.1	9.69	0.82	38.68	26.61	19.25	1.40
November	84.46	15.55	0.35	28.14	6.04	9.80	0.97	38.41	27.73	18.22	1.62
December	85.4	14.6	0.49	27.49	6.06	11.45	1.13	34.71	24.93	18.31	1.51
January	84.3	15.7	0.19	31.31	6.18	11.28	0.99	36.21	34.90	14.45	1.49
Average + STDEV	84.62 ± 1.97	15.38 ± 1.97	0.29 ± 0.08	30.93 ± 2.03	5.44 ± 0.80	10.65 ± 1.62	0.88 ± 0.13	32.73 ± 5.15	25.64 ± 3.49	19.66 ± 2.42	1,44 ± 0.15

Table S4. Composition of various agricultural lignocellulosic biomass outputs.

Lignocellulosic material	Cellulose (wt %)	Hemicellulose (wt %)	Lignin (wt %)	References
Nut shells	25 - 30	25 - 30	30 - 40	[42]
Rice husk	37.1	29.4	24.1	[39]
Sugarcane bagasse	32 - 44	27 - 32	19 - 24	[40]
Cassava peels	37.9 ± 0.33	23.9 ± 0.49	7.5 ± 0.15	[38]
Corn cob	38.27 ± 0.02	46.59 ± 2.5	7.16 ± 0.12	[43]

Sweet sorghum bagasse	45	27	21	[41]
Cocoa husk	25.64 ± 3.49	19.66 ± 2.42	32.73 ± 5.15	This work

Table S5. Proximate composition of sun-dried CH.

Cacao load date	Moisture	Ash	Crude Fat	Crude Protein	Crude Fiber	NFE	Calorific value
				wt %			MJ kg $^{-1}$
February	10.26	10.84	1.00	6.15	35.56	36.19	13.41
March	10.69	11.12	0.57	4.85	37.92	34.85	13.22
April	11.06	9.80	0.67	5.81	33.73	38.93	13.55
May	11.93	8.95	0.92	5.49	31.08	41.63	13.44
June	10.47	8.34	2.33	5.67	30.16	43.03	13.99
July	10.90	10.26	1.46	5.03	32.48	39.87	13.51
August	10.12	9.48	2.58	5.62	29.72	42.48	14.00
September	8.84	11.06	1.04	5.39	29.98	43.69	13.49
October	8.75	10.35	2.51	5.20	27.03	46.16	14.62
November	9.65	13.09	1.42	6.87	31.72	37.25	13.19
December	9.11	11.35	1.57	6.61	33.87	37.49	13.65
January	6.62	12.64	3.27	8.08	29.71	39.68	14.21
Average + STDEV	9.87±1.40	10.61 ±1.39	1.61 ± 0.86	5.90 ± 0.91	31.91 ± 2.98	40.10 ± 3.38	13.69± 0.43

Table S6. Physicochemical composition of CBs.

Load/Date	**Moisture**	**Ash**	**Fat**	**Protein**	**Total Carbohydrates**	**Crude Fiber**	**Total Polyphenols**	**Caloric value**
				wt %			mg EAG g $^{-1}$	MJ kg $^{-1}$
February	4.74	2.93	52.77	12.67	24.67	2.22	49.97	26.51
March	4.53	2.53	54.46	11.34	25.36	2.14	54.54	26.97
April	4.17	2.64	54.16	11.45	24.73	2.64	61.68	26.95
May	3.97	2.51	55.37	12.37	22.66	2.61	36.21	27.25
June	4.48	2.41	55.15	11.98	22.63	3.28	46.80	27.14
July	3.76	2.48	55.05	12.47	23.35	2.89	42.33	27.23
August	3.82	2.24	54.39	11.63	22.64	5.28	42.78	27.12
September	3.79	2.40	53.90	11.85	26.34	2.98	42.52	26.99
October	3.70	2.41	52.68	12.25	26.93	3.34	44.63	26.75
November	4.14	2.57	55.03	12.37	22.39	3.68	52.12	27.15
December	2.94	2.53	55.36	12.31	22.5	4.36	57.58	27.42
January	3.49	2.45	54.62	12.52	23.9	3.02	36.57	27.19
Average+STDEV	3.96 ± 0.50	2.5 ± 0.17	54.41 ± 0.92	12.10 ± 0.44	24.00 ± 2.24	3.20 ± 0.90	47.31 ± 8.03	27.06 ± 0.24

Table S7. Seasonal variations in the proximate analysis of CME from CCN51 cacao fruits.

Load/Date	Analysis results					
	Moisture	Ash	Protein	Total Carbohydrates	Total Polyphenols	Caloric value
			wt %		mg EAG g^{-1}	MJ kg g^{-1}
February	71.59	0.53	0.43	27.45	0.24	4.67
March	71.52	0.61	0.39	29.26	0.29	4.96
April	80.78	0.46	0.36	22.67	0.13	3.85
May	79.7	0.45	0.34	19.5	0.43	3.32
June	80.34	0.43	0.32	19.21	0.77	3.27
July	84.77	0.42	0.33	14.48	0.4	2.48
August	88.63	0.48	0.25	10.91	0.42	2.15
September	88.27	0.32	0.23	12.6	0.4	2.15
Octuber	86.33	0.33	0.24	13.09	0.35	2.23
November	87.21	0.27	0.21	11.7	0.31	1.99
December	84.32	0.57	0.17	14.94	1.17	2.53
January	88.64	0.54	0.46	10.36	1.7	1.81
Average+STDEV	82.97 ± 6.09	0.45 ± 0.10	0.31 ± 0.09	17.18 ± 6.44	0.55 ± 0.45	2.95 ± 1.06

Table S8. Physicochemical analysis of CME.

Cacao load date	Saccharose	Glucose	Fructose	Total sugars	Total soluble solids °Brix	Density	pH	Total solids	Sodium	Aluminum	Calcium	Potassium
February	8.06	68.34	78.75	155.15	9.20	1.04	3.72	15.75	NR	NR	NR	NR
March	11.70	72.84	83.11	167.65	10.60	1.04	3.76	16.59	NR	NR	NR	NR
April	6.84	74.20	79.38	160.42	17.00	1.07	3.74	14.92	0.85	4.68	42.56	2623.15
May	9.27	73.52	81.24	164.04	12.00	1.05	3.80	18.25	NR	NR	NR	NR
June	8.59	58.94	84.43	151.96	13.00	1.06	3.71	17.44	NR	NR	NR	NR
July	8.93	66.23	82.84	158.00	7.00	1.03	3.85	17.85	NR	NR	NR	NR
August	8.10	73.56	82.17	163.83	6.00	1.02	3.80	17.33	NR	NR	NR	NR
September	7.27	80.89	81.50	169.66	15.00	1.03	3.94	17.11	1.13	6.32	68.18	2074.51
October	20.50	67.59	70.40	158.49	12.40	1.06	3.81	17.56	1.29	7.31	60.90	2943.52
November	33.73	54.30	59.30	147.33	12.00	1.05	3.89	16.34	0.98	5.33	57.82	2059.45
December	26.58	56.55	60.89	144.02	12.20	1.05	3.85	16.95	1.13	6.32	59.36	2501.49
January	19.43	58.80	62.48	140.71	12.10	1.05	3.87	16.64	1.05	5.82	58.59	2280.47
Average±STDEV	14.08 ± 8.89	67.15 ± 8.36	75.54 ± 9.54	156.77 ±9.25	11.54 ± 3.07	1.05 ±0.01	3.81 ± 0.07	16.89 ± 0.92	1.07 ± 0.15	5.96 ± 0.91	57.9 ± 8.40	2413.77 ± 343.68
